# Efficacy of Ketamine in the Treatment of Substance Use Disorders: A Systematic Review

**DOI:** 10.3389/fpsyt.2018.00277

**Published:** 2018-07-24

**Authors:** Jennifer L. Jones, Camilo F. Mateus, Robert J. Malcolm, Kathleen T. Brady, Sudie E. Back

**Affiliations:** ^1^Medical University of South Carolina, Charleston, SC, United States; ^2^Ralph H. Johnson VA Medical Center, Charleston, SC, United States

**Keywords:** ketamine, substance use disorders, addiction, glutamate, abstinence

## Abstract

**Background:** Despite advances in behavioral and pharmacotherapy interventions, substance use disorders (SUDs) are frequently refractory to treatment. Glutamatergic dysregulation has received increasing attention as one common neuropathology across multiple substances of abuse. Ketamine is a potent N-methyl-D-aspartate (NMDA) glutamatergic receptor antagonist which has been found to be effective in the treatment of severe depression. Here we review the literature on the efficacy of ketamine in the treatment of SUDs.

**Methods:** A systematic review of the PubMed, Scopus, and ClinicalTrials.gov databases was undertaken to identify completed and ongoing human studies of the effectiveness of ketamine in the treatment of SUDs between January 1997 and January 2018.

**Results and conclusion:** Seven completed studies were identified. Two studies focused on alcohol use disorder, two focused on cocaine use disorder, and three focused on opioid use disorder. Both cocaine studies found improvements in craving, motivation, and decreased cocaine use rates, although studies were limited by small sample sizes, a homogeneous population and short follow-up. Studies of alcohol and opioid use disorders found improvement in abstinence rates in the ketamine group, with significant between-group effects noted for up to two years following a single infusion, although these were not placebo-controlled trials. These results suggest that ketamine may facilitate abstinence across multiple substances of abuse and warrants broader investigation in addiction treatment. We conclude with an overview of the six ongoing studies of ketamine in the treatment of alcohol, cocaine, cannabis, and opioid use disorders and discuss future directions in this emerging area of research.

## Introduction

Alcohol and illicit drug use is an escalating and complex global public health burden. In 2010, the global prevalence of alcohol and illicit drug use disorders were 9.6 and 10.9% respectively (1). Mortality rates have risen to epidemic proportions in some countries due to increasing prevalence of opioid use. For example, the United States, which accounts for 25% of global overdose mortality, has experienced an 88% increase in opioid overdose deaths each year from 2013 to 2016 (2, 3). Substance use disorders (SUDs) include cognitive, behavioral, and physiological symptoms. Hallmark signs of SUDs include impaired control, cravings, social impairment, risky use, and withdrawal symptoms. Withdrawal from heavy, prolonged alcohol use can result in life-threatening seizures and autonomic instability in addition to hallucinations, severe agitation, and anxiety. Physiologic response to opioid withdrawal can also be severe, and includes nausea, emesis, diarrhea, myalgias, intractable lacrimation and rhinorrhea, fevers, dysphoria and insomnia. Fear of these withdrawal symptoms is frequently cited as a barrier to treatment and reason for relapse (4).

Despite the high prevalence and substantial societal burden of SUDs, effective pharmacotherapy options are limited. FDA-approved medications for alcohol use disorder include naltrexone (an opioid receptor antagonist) and acamprosate (a GABA-agonist) which have been shown in meta-analyses to modestly reduce rates of return to heavy drinking (5), and disulfiram and nalmefene (approved in the European Union although not FDA-approved in the U.S.) have shown overall Hedge's g effect sizes of 0.58 and 0.33, respectively (6, 7). Treatment options for opioid dependence include full opioid agonists (methadone), partial agonists (buprenorphine) and antagonists (naltrexone). For cannabis and stimulant use disorders, there are no FDA-approved treatments (8). With limited treatment options, a myriad of non-FDA approved medications (e.g., gabapentin, clonidine, bupropion) are tried as standalone pharmacotherapies and in conjunction with behavioral interventions.

Glutamatergic dysregulation in the prefrontal cortex and mesolimbic regions (including the amygdala and the nucleus accumbens) has been implicated in addiction pathology across multiple substances of abuse (9). Similarly, depression has been shown to have aberrant glutamate signaling (10–12). Ketamine is a potent, non-competitive NMDA receptor antagonist which has been widely used in conjunction with general anesthesia following FDA approval in the U.S. in 1970. More recently, ketamine has been shown in two meta-analyses to induce ultra-rapid remission of severe depression and suicidal ideation using sub-anesthetic dosages (13–15). This anti-depressant effect is hypothesized to result from improved prefrontal cortex glutamate homeostasis (16). These changes ultimately produce synaptic improvements such as structurally increased spine density at synaptic proteins (17). These effects may improve ability to learn new behaviors (18) and may be beneficial in the treatment of SUDs. Our overall objective is to provide a review of the recent literature on the efficacy of ketamine in the treatment of SUDs.

## Methods

**Figure 1:**
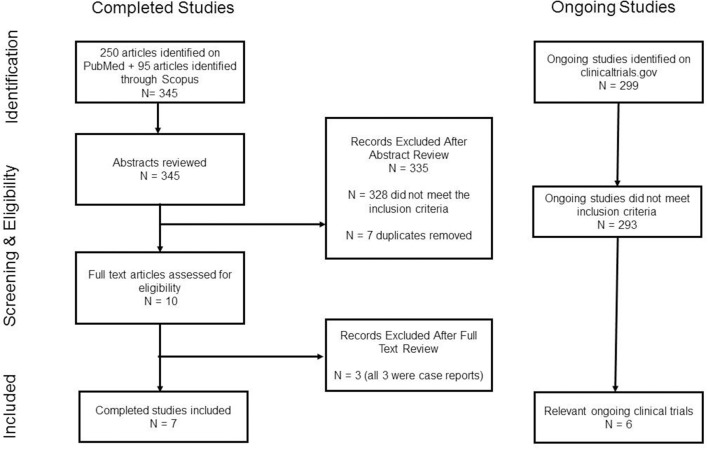
Study flow diagram.

A comprehensive search in the PubMed/MEDLINE, Scopus and clinicaltrials.gov databases from 1 January 1996 to 1 January 2018 was conducted. PubMed was searched using the following MESH search terms: “Substance-Related Disorders” and “Ketamine/therapeutic use.” The Scopus database was searched using the term “ketamine” in conjunction with the following title keywords: “cocaine,” “alcohol,” “cannabis,” “marijuana,” “amphetamine,” “methamphetamine,” “tobacco,” “nicotine,” “heroin,” and “opi.^*^” Results from both databases were filtered to include only human studies with full text articles available in English. Case reports were excluded. “Ketamine” was the term used in the clinicaltrial.gov database search of active studies. The inclusion criteria were as follows: studies must have evaluated the efficacy of ketamine (with or without a behavioral intervention) in humans for the treatment of a SUD or the treatment of withdrawal symptoms from a substance of abuse. See Figure 1 for detailed study methodology characteristics.

## Results

After evaluating for the inclusion criteria and for duplicates, we identified seven completed relevant clinical studies. Six ongoing relevant clinical trials were also identified. Substances of abuse studied included cocaine, alcohol, and opioids. No human studies were found that evaluated the efficacy of ketamine in the treatment of tobacco or stimulant use disorders other than cocaine. The included studies are summarized in Table 1.

**Table 1:** Characteristics of the included studies.

**References**	**Substance of abuse and sample characteristics**	**Ketamine/control intervention**	**Main outcome measures**	**Results**	**Study limitations**
**COMPLETED STUDIES**					
(19)	Cocaine 8 non-treatment and non-abstinence seeking participants aged 21–52, meeting DSM-IV criteria for cocaine dependence.	Three arm, cross-over design RCT in which participants were hospitalized for 9 days, undergoing infusions of 0.41 mg/kg ketamine, 0.71 mg/kg ketamine, and 2 mg lorazepam at intervals of 48 h, with assessments performed 24 h post-infusion.	Motivation levels to stop cocaine use (assessed using the URICA) and levels of cue-induced craving (using the VAS).	Motivation to quit trended toward significance (*p* = 0.05) with a median improvement in URICA scores from 0.71 to 4.87. There was a significant reduction in cocaine use compared to baseline as quantified by median amount ($149.30/using day at baseline vs. $10.50 post-treatment, *p* < 0.001) and frequency (22/28 using day pre-treatment, compared to 5/28 post-treatment, *p* = 0.01).	Small sample size with narrow demographic profile (55–87.5% male, 75–87.5% African American) limits extensive external comparisons. Cross-over design limits ability to analyze duration of effects.
(20)	Cocaine 20 non-depressed, non-treatment seeking participants aged 21–55, meeting DSM-IV criteria for cocaine dependence as well as a minimum criteria for use (at least 2 days of cocaine use per week, spending more than $40 per use).	Two arm, cross-over design RCT in which participants were hospitalized on 3 occasions at 2 week intervals and underwent choice sessions during which they were offered either 25 mg cocaine or $11. Baseline rates were established using a saline infusion during hospitalization #1. During hospitalizations #2 and #3, participants underwent either ketamine infusion (0.71 mg/kg) or midazolam (0.025 mg/kg) 28 h prior to the choice session.	Number of cocaine self-administration choices at 28 h post-infusion relative to baseline. Secondary outcome measures included cocaine use rates following discharge #2 and stress reactivity scores (measured using the FFMQ).	Self-administration rates were reduced significantly in the ketamine group (1.61 cocaine choices vs. 4.33 in the midazolam group, *p* < 0.0001). Non-reactivity rates were significantly improved in the ketamine group at 3.46 (vs. 2.92 in the midazolam group, *p* < 0.05). Ketamine use was significantly reduced in the acute time period (1–6 days) post discharge ($22.45 vs. $3.20, *p* < 0.05), but lost significance over longer follow-up. Additionally, 2/10 participants were voluntarily abstinent in the 2 weeks post-discharge in the ketamine group, compared to 0/10 in the midazolam group. Craving rates were significantly reduced at time of discharge by 59.6% in the ketamine group compared to 15.3% in the midazolam group, although this lost significance at subsequent follow-up time points.	Small sample size with narrow demographic profile limits extensive external comparisons. Cross-over design limits ability to analyze duration of effects.
(21)	Opioids 70 participants aged 18–30 meeting DSM-IV criteria for heroin dependence who had recently undergone a 2-week inpatient detoxification.	Two arm RCT in which participants were randomized to a single session of high dose (2 mg/kg) or low dose (0.2 mg/kg) IM ketamine in conjunction with existentially-oriented psychotherapy.	Self-reported abstinence rates following discharge assessed on a monthly basis for 2 years.	One and two year complete heroin abstinence rates of 24% and 17% in the high dose group vs. 6 and 2% in the low dose group, *p* < 0.05).	Lack of clarity regarding the event of possible discordance in abstinence outcomes suggests possible reporting bias. No placebo group (only low dose comparison group). No follow-up on opioid substitution pharmacotherapy.
(22)	Opioids 59 participants aged 18–35 meeting DSM-IV criteria for heroin dependence who had recently undergone a 2-week inpatient detoxification	Two arm RCT in which all participants underwent existentially-oriented psychotherapy in conjunction with 2 mg/kg IM ketamine. Participants were then randomized to either 2 general addiction counseling sessions on a monthly interval or 2 additional ketamine-guided psychotherapy sessions at a dose of 2 mg/kg IM.	Self-reported abstinence rates following discharge assessed on a monthly basis for 2 years.	At 1 year follow-up, 13/26 (50%) of participants in the multiple ketamine infusion group remained fully abstinent from heroin vs. 6/27 (22.2%) subjects in the single session group.	No placebo group (only low dose comparison group). No follow-up on opioid substitution pharmacotherapy.
(23)	Opioids 58 participants aged 18–35 meeting DSM-IV criteria for opioid dependence.	Two arm RCT in which all participants underwent general anesthesia for rapid induction of opioid withdrawal. Ketamine (0.5 mg/kg/h) vs. saline placebo were compared as part of maintenance of anesthesia prior to opiate antagonist (naltrexone) induction while under general anesthesia.	Cardiovascular response (blood pressure, pulse), respirations, renal output and gastrointestinal output as well as cortisol levels were measured. Subjective and Objective Opiate Withdrawal Scales (SOWS and OOWS) were assessed during the early post-anesthetic period. Abstinence rates at 4 months were also secondarily assessed.	The ketamine group showed significant lower increases in blood pressure, pulse and cortisol levels during rapid opiate antagonist induction under general anesthesia. The ketamine group had lower OOWS at hours 1 and 2 of during the anesthesia phase. In the early post-anesthesia phase, patients in the ketamine group required less adjuvant carbamazepine and clonazepam to maintain equivalent opioid withdrawal symptoms compared to the placebo group (*p* < 0.001). There were no significant differences in terms of abstinence rates at 4-month follow-up.	Subsequent opioid antagonist use in the follow-up phase was not assessed. Abstinence rates were not tracked in interim follow-up, limiting the discernment of duration of effects.
(24)	Alcohol 111 treatment-seeking alcohol dependent participants and 100 treatment seeking controls, aged 23–56.	Two arm, case-control study in which participants initially completed a 3 month inpatient detoxification program. The ketamine group then underwent a single session of 2.5 mg/kg IM ketamine in conjunction with existentially oriented psychotherapy. The control group received routine follow-up.	Self-reported abstinence rates following discharge assessed on a monthly basis for 2 years.	Total abstinence rates at 1 year follow-up of 73/111 (65.8%) in the ketamine treatment group as compared to 24/100 (24%) in the treatment as usual group. Two year follow-up rates of total abstinence were 40.7% in the ketamine group, although comparisons could not be made to the control group, which was only followed for a 1 year interval.	Study methodology suggests potential risk of selection bias. The addition of a behavioral intervention (existential psychotherapy) for the treatment but not comparison group limits isolation of ketamine's effects.
(25)	Alcohol 23 patients aged 47–54 admitted to a medical ICU for management of alcohol withdrawal syndrome.	Retrospective review of 23 patients who were admitted for management of severe alcohol withdrawal symptoms and administered ketamine as an adjunct to conventional treatment with benzodiazepines.	Median change in benzodiazepine requirements to maintain Withdrawal Assessment Scale values < 10, frequency of delirium tremens and other alcohol withdrawal symptoms.	While 75% of patients developed symptoms of delirium tremens early in their hospitalization, no AWS complications (delirium tremens, hallucinations, or seizures) occurred after ketamine was initiated. There was a trend toward decreased benzodiazepine requirements at 12 and 24 h after initiation of ketamine infusion with median changes of −40 mg and −13.3 of diazepam equivalents (*p* = 0.11 and *p* = 0.33, respectively).	Retrospective review in which ketamine infusions were not standardized in dose or frequency. Adjunctive anesthesia agents were not standardized between patients, and patients were neither randomized nor matched on withdrawal severity.
**ONGOING STUDIES**					
Ketamine for Reduction of Alcoholic Relapse/Celia Morgan, PhD	Alcohol	Estimated enrollment of 96 recently detoxified participants, ages 18-60, meeting DSM-V criteria for alcohol use disorder and minimum of mild depression (BDI-II score >14).	RCT with 4 groups, comparing ketamine with saline placebo and manualized relapse prevention psychotherapy with simple education about alcohol effects. Both groups will also receive 12 weeks of motivation enhancement therapy and 4 weeks of mindfulness based relapse prevention.	Arm 1: 0.8 mg/kg IV ketamine + manualized relapse prevention psychotherapy (RPT). Arm 2: a 0.8 mg/kg IV ketamine session + simple alcohol education (AE). Arm 3: saline placebo + RPT. Arm 4: saline placebo + AE.	Primary outcome measures are abstinence rates at 3 and 6 months, as well as relapse rates to alcohol at 6 months. Secondary outcome measures include depressive symptoms, craving, and quality of life.
Glutamatergic Modulation of Disordered Alcohol Use /Elias Dakwar, MD	Alcohol	Estimated enrollment of 40 treatment seeking participants, ages 21-69, meeting DSM-IV criteria for active alcohol dependence.	RCT with 2 groups, both groups also receive 5 weeks of motivational enhancement therapy.	Arm 1: 0.71 mg/kg IV ketamine in wk2 Arm 2:0.025 mg/kg ketamine IV in wk2.	Primary outcome measure will be change from baseline to wk 5 in # drinking days (TLFB).
Ketamine for the Rapid Treatment of Major Depressive Disorder and Alcohol Use Disorder/Gihyun Yoon, MD	Alcohol	Estimated enrollment of 65 participants, ages 21–65 meeting DSM-V criteria for MDD and AUD.	RCT with 3 groups. All groups will receive weekly infusions of ketamine or saline placebo. Participants will also receive 2 intramuscular (IM) injections of either naltrexone or saline placebo.	Arm 1: 0.5 mg/kg IV ketamine weekly × 4 wks + monthly IM naltrexone (380 mg). Arm 2: 0.5 mg/kg IV ketamine weekly × 4 wks + monthly IM saline placebo. Arm 3: psychoactive placebo 0.045 mg/kg midazolam) + Placebo weekly × 4 weeks.	Primary outcome measures will be: depression severity from baseline to day 21 (MADRS) and rate of complete abstinence from alcohol from baseline to day 21 (TLFB).
Glutamatergic Modulation to Facilitate the Behavioral Treatment of Cocaine Use Disorders/Elias Dakwar, MD	Cocaine	Estimated enrollment of 150 treatment-seeking participants, ages 21–60, meeting DSM-V criteria for cocaine use disorder.	RCT with 2 groups, both groups also receive 12 weeks of MET and 4 weeks of MBRP.	Arm 1: 0.71 mg/kg IV ketamine in wk1+ wk5. Arm 2: 0.025 mg/kg ketamine IV in wk1+ wk5.	Primary outcome measure is abstinence from cocaine use from baseline to wk12 (TLFB).
Facilitating Rapid Naltrexone Initiation/Elias Dakwar, MD	Opioids	Estimated enrollment of 100 treatment-seeking participants, ages 18–60, undergoing acute inpatient detoxification over the course of 5 days.	RCT with 2 groups, both groups also receive 12 weeks of mindfulness based relapse prevention and motivational interviewing. Participants will undergo an initial ketamine infusion when they begin experiencing moderate withdrawal and be up-titrated on oral naltrexone as tolerated during hospitalization.	Arm 1: Two infusions of 0.11 mg/kg IV ketamine bolus + 1.3 mg/kg IV ketamine over 90 min, over the course of 2 consecutive days. Arm 2: Two infusions of saline bolus + 0.0125 mg/kg IV ketamine over 90 min over the course of 2 consecutive days.	Primary outcome will be rates of initiation on extended-release naltrexone.
Facilitating the Behavioral Treatment of Cannabis Use Disorder/Elias Dakwar, MD	Cannabis	Estimated enrollment of 15 treatment seeking participants, ages 21–60, meeting DSM-IV criteria for cannabis dependence.	Single-blind trial, with participants given the understanding that they may receive any of several medications in conjunction with a 2 week course of MET and then a 4 week course of MBRP.	Arm 1: Initial infusion of 0.71 mg/kg IV ketamine in week 2 + possible second infusion in week 3 or 4.	Primary outcome will be rates of cannabis use from baseline to week 6 (UDS).

*RCT, Randomized controlled trial; URICA, University of Rhode Island Change Assessment; VAS, Visual Analog Scale; FFMQ, Five Facet Mindfulness Questionnaire; BDI-2, Beck's Depression Inventory-II; TLFB, Timeline Followback; MADRS, Montgomery–Asberg Depression Rating Scale; RPT, Relapse prevention psychotherapy; AE, Alcohol education; UDS, Urine drug screen; MET, Motivational enhancement therapy; MBRP, Mindfulness based relapse prevention*.

### Effects on cocaine use

Two published studies have evaluated the efficacy of ketamine for cocaine use disorder. Dakwar et al. (19) conducted a three-arm crossover trial which evaluated the effects of 0.41 and 0.71 mg/kg IV ketamine compared to an active placebo control (2 mg lorazepam) in eight non-treatment seeking cocaine dependent participants. Low dose ketamine always preceded the high dose and infusions were spaced at 48 h intervals. Primary outcome measures were changes in pre-infusion and 24-h post-infusion levels of motivation to quit cocaine as assessed by the University of Rhode Island Change Assessment (URICA) questionnaire and self-reported cocaine cravings on the Visual Analog Scale (VAS). VAS measurements were taken every 3 min during a 15-min cocaine cue exposure task, with a total score range of 0–600 mm. Within-subject statistical comparisons were made to baseline scores to assess order effects. Following the first infusion (either 0.41 mg/kg ketamine or 2 mg lorazepam), they found that ketamine increased motivation to quit cocaine over lorazepam (median score of 0.15 vs. 3.6, *p* = 0.012) and reduced cocaine craving on the VAS by a mean of 168 mm (a 60% change, *p* = 0.012). The post-lorazepam URICA scores were significantly improved when ketamine was received first (median change from baseline: 1.1 vs. 3.28, *p* = 0.047), indicative of a persistent effect of 0.41 mg/kg ketamine at 48 h; this may explain the lack of significant change in URICA and VAS scores following the 0.71 mg/kg ketamine infusion. Although there was no placebo comparison, there was a significant reduction in frequency (22 days of use/28 days at baseline vs. 5/28 days at 4 week follow-up, *p* = 0.012) and amount of cocaine use in the follow-up period ($149.30/use day at baseline vs. $10.50/use day at 4 week follow-up, *p* < 0.001).

In a related follow-up study of 20 non-treatment seeking cocaine dependent participants, Dakwar et al. (20) conducted a cross-over design, inpatient laboratory paradigm trial evaluating the efficacy of a single infusion of 0.71 mg/kg ketamine with 0.025 mg/kg midazolam as the active control. During a 6-day hospitalization, subjects participated in “choice sessions” (during which they could elect to self-administer 25 mg cocaine or receive $11). Rates of cocaine self-administration were reduced by 66% relative to pre-infusion baseline choice rates with no significant pre-/post- infusion self-administration differences noted in the control group (*p* < 0.0001).

### Effects on opioid use disorder and opioid withdrawal

Two published studies have evaluated the efficacy of ketamine for opioid use disorder. Krupitsky et al. (21) conducted a randomized controlled trial of 70 heroin-dependent participants in which they compared the efficacy of high dose ketamine (2 mg/kg IM) vs. low dose ketamine (0.2 mg/kg IM) in conjunction with psychotherapy. Primary outcome measures were heroin abstinence rates (assessed by self-report, collateral information, physical examination of skin, and urine drug screen) at 1, 3, 6, 12, 18, and 24 months. Abstinence rates at 1 month approached 85% in the 2 mg/kg group (compared to 55% in the 0.2 mg/kg group, *p* < 0.01) and were 24% at 1 year in the 2 mg/kg group (compared to 6% in the 0.2 mg/kg group, *p* < 0.05). Craving was also notably reduced in the high vs. low groups, with an enduring decline craving noted pre-/post- infusion in the high dose group on the VAS (baseline mean of 29.24 mm, 7.7 mm at 1 month, and 5.4 mm at 3 month follow-up, *p* < 0.001). In a follow-up study, Krupitsky et al. (22) evaluated the efficacy of single vs. repeated sessions of ketamine-assisted psychotherapy in increasing abstinence from heroin. Participants were randomized to a 1 or 3 sessions of ketamine (2 mg/kg IM, given at 1 month intervals). They found that 50% of subjects receiving multiple ketamine treatments were abstinent at 1 year follow-up, compared to 22% of single-session treatments (*p* < 0.05). They also noted significantly greater reductions in heroin craving in the repeated treatment group as compared to the single treatment group.

Jovaiša et al. (23) conducted a randomized controlled trial in which participants were given either saline placebo infusion or 0.5 mg/kg/h of IV ketamine prior to rapid opiate antagonist induction under general anesthesia. Their results showed that ketamine could suppress physiologic response to opiate withdrawal. Mean arterial pressure, heart rate, and serum cortisol were significantly lower in the ketamine group during opiate antagonist induction under anesthesia. There were no significant group differences at 4 months on their secondary outcome measures of aftercare treatment retention, abstinence rates, self-reported health, or social/family life improvements, although both the placebo and ketamine groups were also started on opioid antagonist treatment. This lack of group differences may be related to initial opioid antagonist treatment in both groups or to administration of ketamine while the participants were unconscious.

### Effects on alcohol use disorder and alcohol withdrawal symptoms

Krupitsky et al. (24) evaluated two cohorts with alcohol use disorder that underwent inpatient detoxification and residential treatment. Following the 3-month residential treatment, the group compared the abstinence rates of 111 participants who volunteered to undergo a ketamine-assisted psychotherapy session with 100 subjects who completed only follow-up as usual. They found that 1-year abstinence rates were 65.8% in the ketamine-treated group compared to 24% in the follow-up as usual comparison group (*p* < 0.01). However, the study was not randomized and is limited by the lack of a control group. Wong et al. (25) completed a retrospective review of 23 patients who were hospitalized for management of severe alcohol withdrawal symptoms and who were administered ketamine as an adjunct to conventional treatment with benzodiazepines. They found a trend (*p* = 0.11) toward reduced benzodiazepine requirements at 12 and 24 h post-ketamine initiation with medians of −40 and −13.3 mg of diazepam equivalents.

### Ongoing studies

Six ongoing studies were identified through clinical trials.gov that are evaluating the use of ketamine in the treatment of SUDs (see Table 1). Three of these studies are focused on alcohol use disorder, and the other three are focused on cocaine, opioid, and cannabis use disorders.

The first randomized controlled trial evaluating the efficacy of ketamine in alcohol use disorder is in progress (NCT02649231 led by Celia Morgan, Ph.D.). This trial of 96 participants will compare ketamine with saline placebo and manualized relapse prevention psychotherapy with simple education about alcohol effects. Primary outcome measures include abstinence rates at 3 and 6 months, and relapse rates at 6 months. This study will evaluate the efficacy of a 0.8 mg/kg ketamine infusion in maintenance of abstinence from alcohol. By studying the effects on depression as a secondary outcome, this study will add to the intriguing findings that a positive family history of alcohol use disorder is associated with greater duration of anti-depressant effect (13). The inclusion of simple alcohol education and manualized relapse prevention psychotherapy arms will also assess whether the effects of ketamine are enhanced with cognitive behavioral psychotherapy.

A related randomized controlled trial of 40 subjects (NCT02539511 led by Elias Dakwar, MD) will evaluate the efficacy of a single infusion of ketamine (0.71 mg/kg) in conjunction with a 5 week course of motivational enhancement psychotherapy in reduction in alcohol use. Primary outcome measures include change in self-reported alcohol use rates at 5 weeks vs. baseline. These results will provide complementary information as to the efficacy of ketamine in conjunction with a standardized psychotherapy.

A third study of 65 subjects with alcohol use disorder and major depressive disorder (NCT02461927 led by Gihyn Yoon, MD) will evaluate the effects of ketamine in 3 treatment arms (0.5 mg/kg ketamine + 380 mg IM naltrexone vs. 0.5 mg/kg ketamine alone vs. placebo). The ketamine treatments will consist of once weekly infusions of IV ketamine for a total of 4 weeks and two injections of naltrexone or saline placebo spaced 1 month apart. Primary outcome measures include change in depression severity as measured by the Montgomery-Asberg Depression Rating Scale and rate of complete abstinence from alcohol as measured on the Time Line Follow Back at 4 week follow-up. This trial will inform questions regarding the utility of weekly infusion sessions as well as combination pharmacotherapy with naltrexone.

In a follow-up study to his initial investigations of ketamine in cocaine use disorder, Elias Dakwar, MD is leading a randomized, placebo-controlled trial (NCT03344419) of 150 subjects to evaluate the efficacy of 2 ketamine infusions (0.71 mg/kg active dose at a 1 month interval) on abstinence rates at baseline and following 12 weeks of adjunctive psychotherapy. This study will expand on the prior findings that ketamine can increase ability to achieve and maintain abstinence from cocaine and further evaluate the duration of efficacy in cocaine abstinence.

A related study (NCT03345173 led by Elias Dakwar, MD) will evaluate the use of ketamine in the acute detoxification of 100 opioid users to facilitate transition to extended release naltrexone injections. Participants will initially be hospitalized for up to 5 days for detoxification followed by naltrexone initiation (with ketamine infusions of 0.11 mg/kg in a 2-min bolus followed by 1.3 mg/kg over 90 min on 2 sequential days when they begin experiencing moderate withdrawal symptoms). Subjects will then complete 12 weeks of motivational enhancement therapy and mindfulness based relapse prevention psychotherapy. Primary outcome measure will be rates of successful initiation on extended release naltrexone.

A 15 subject, proof-of-concept study (NCT02946489 led by Elias Dakwar, MD) will investigate the use of 0.71 mg/kg ketamine in cannabis use disorder. This randomized controlled trial is the first to explore the effects of ketamine on cannabis abstinence rates. This study will recruit treatment seeking individuals and will also include motivational enhancement therapy and mindfulness based relapse prevention psychotherapy components. Abstinence rates will be assessed at baseline and at 6 week follow-up.

## Discussion and future directions

Collectively, these studies suggest that ketamine may improve the ability to establish and maintain abstinence in SUDs. Improvement in cravings, motivation to quit, and self-administration have been shown in cocaine use disorder (19, 20, 26). Significant long-term improvements in complete abstinence from alcohol and heroin have been demonstrated with ketamine following extended inpatient treatment (21, 22, 24), and ketamine reduced physiological response during opioid withdrawal (23). However, these preliminary studies have several important limitations. The findings in the cocaine trials are limited by small sample sizes, narrow demographic sectors, and limited follow-up windows (19, 20). Additionally, both the heroin and alcohol use disorder studies by Krupitsky et al. (21), Krupitsky et al. (22), and Jovaiša et al. (23) utilized a low dose ketamine comparison group rather than a true placebo control and did not control for adjunctive pharmacotherapy in the follow-up.

A number of important questions also remain. It is unclear to what extent baseline motivation, desire to quit, or duration of prior abstinence influences the effectiveness of ketamine in achieving and maintaining abstinence. The heroin and alcohol dependent populations in Russia studied (21, 22, 24) were treatment seeking and had completed 3 months of residential inpatient treatment prior to ketamine infusions; this is markedly different from the non-treatment seeking cocaine studies (19, 20). It is of note however, that 20% of the non-treatment, non-abstinence seeking cocaine trial participants (19) were voluntarily abstinent following the single ketamine infusion (compared to 0% of the midazolam control group). While the abstinence improvements in heroin use noted at 1 and 2 year follow-up are promising (21, 22), their unique demographic, genetic, and socioeconomic characteristics may contribute to these results. Potential gender differences are also an important aspect to consider in future trial design and analysis.

The effects of ketamine on withdrawal states are particularly important to further investigate. Alcohol and benzodiazepine withdrawal can result in life-threatening medical sequela, and the severe physiologic response to opioid withdrawal may deterrent to initial treatment. The opioid withdrawal study (23) showed physiologic suppression of opiate withdrawal. While the effects of ketamine on opioid withdrawal independent from its use in conjunction with general anesthesia have not been systematically studied, several case reports have utilized ketamine in conscious patients with improvement in their opioid withdrawal symptoms (27–29). The results from the ongoing naltrexone induction study should provide some insight as to whether ketamine improves opioid withdrawal symptoms independent from rapid opioid induction under general anesthesia. Future studies should assess and report on concurrent use of FDA-approved treatments for opioid use disorder (both at baseline and in the follow-up phase). Prospective trials are also needed to give further information about ketamine's efficacy in alcohol withdrawal.

The utility of behavioral interventions as adjuvants to ketamine pharmacotherapy in addictions treatment is understudied. The ongoing trial led by Morgan and colleagues will evaluate the addition of psychotherapy to ketamine infusions. However, additional research is needed to examine behavioral interventions which may be synergistic with ketamine pharmacotherapy and help enhance long-term treatment outcomes.

At sub-anesthetic dosing, ketamine produces mild dissociative psychoactive effects (30, 31). While these psychotomimetic characteristics may increase abuse liability (32), more recent studies in both depression and substance abuse populations suggest that the therapeutic events of ketamine may be mediated by participant perception of these psychoactive effects (26, 30, 31). Future studies should include assessment of the psychoactive effects of ketamine to further evaluate whether perceptual experience mediates therapeutic benefit.

Finally, future ketamine trials should include evaluation of optimal dose and frequency schedules. The majority of the studies have utilized prior depression trial dosages of 0.5–0.8 mg/kg IV ketamine, although a few studies utilized doses of 2–2.5 mg/kg IM. Intranasal dosing (which is currently under evaluation for the treatment of depression) could also widely expand the availability of ketamine treatment. Further characterization in other substances of abuse (such as nicotine, amphetamines, and the ongoing cannabis trial) may also provide important insights as to the overall efficacy of ketamine in the treatment of SUDs. In summary, the most pressing public health question is whether ketamine (in single or multiple dose treatments) can significantly reduce addiction morbidity and mortality. Further studies are urgently needed.

## Author contributions

JJ designed the strategy for the present review, searched for the references, read the manuscripts, and drafted the manuscript. CM drafted content for the manuscript introduction and figure. RM, KB, and SB provided content and editorial oversight. All authors discussed the results, reviewed the manuscript, and helped with the final writing.

### Conflict of interest statement

The authors declare that the research was conducted in the absence of any commercial or financial relationships that could be construed as a potential conflict of interest.
